# Traumatic Brain Injury Induces Microglial and Caspase3 Activation in the Retina

**DOI:** 10.3390/ijms24054451

**Published:** 2023-02-23

**Authors:** Tamás Kovács-Öller, Renáta Zempléni, Boglárka Balogh, Gergely Szarka, Bálint Fazekas, Ádám J. Tengölics, Krisztina Amrein, Endre Czeiter, István Hernádi, András Büki, Béla Völgyi

**Affiliations:** 1 Retinal Neurobiology Research Group, Szentágothai Research Centre, University of Pécs, 7624 Pécs, Hungary; 2 Institute of Biology, Faculty of Sciences, University of Pécs, 7624 Pécs, Hungary; 3 Neurotrauma Research Group, Szentágothai Research Centre, University of Pécs, 7624 Pécs, Hungary; 4 Department of Neurosurgery, Medical School, University of Pécs, 7623 Pécs, Hungary; 5 Department of Biological and Visual Sciences, SUNY College of Optometry, New York, NY 10036, USA; 6 ELKH-PTE Clinical Neuroscience MR Research Group, University of Pécs, 7623 Pécs, Hungary; 7 Grastyán Translational Research Center, University of Pécs, 7624 Pécs, Hungary; 8 Department of Neurosurgery, School of Medical Sciences, Faculty of Medicine and Health, Örebro University, 701 85 Örebro, Sweden

**Keywords:** TBI, brain, injury, microglia, caspase, apoptosis, retina, degeneration

## Abstract

Traumatic brain injury (TBI) is among the main causes of sudden death after head trauma. These injuries can result in severe degeneration and neuronal cell death in the CNS, including the retina, which is a crucial part of the brain responsible for perceiving and transmitting visual information. The long-term effects of mild–repetitive TBI (rmTBI) are far less studied thus far, even though damage induced by repetitive injuries occurring in the brain is more common, especially amongst athletes. rmTBI can also have a detrimental effect on the retina and the pathophysiology of these injuries is likely to differ from severe TBI (sTBI) retinal injury. Here, we show how rmTBI and sTBI can differentially affect the retina. Our results indicate an increase in the number of activated microglial cells and Caspase3-positive cells in the retina in both traumatic models, suggesting a rise in the level of inflammation and cell death after TBI. The pattern of microglial activation appears distributed and widespread but differs amongst the various retinal layers. sTBI induced microglial activation in both the superficial and deep retinal layers. In contrast to sTBI, no significant change occurred following the repetitive mild injury in the superficial layer, only the deep layer (spanning from the inner nuclear layer to the outer plexiform layer) shows microglial activation. This difference suggests that alternate response mechanisms play a role in the case of the different TBI incidents. The Caspase3 activation pattern showed a uniform increase in both the superficial and deep layers of the retina. This suggests a different action in the course of the disease in sTBI and rmTBI models and points to the need for new diagnostic procedures. Our present results suggest that the retina might serve as such a model of head injuries since the retinal tissue reacts to both forms of TBI and is the most accessible part of the human brain.

## 1. Introduction

The term traumatic brain injury (TBI) is used as a collective term for pathological changes due to external forces that can lead to physiological, cognitive, and psychosocial disorders of the central nervous system. It is most commonly caused by accidents, sports injuries, and falls [1]. These injuries can result in a variety of disorders, disabilities, and sometimes seizures [2]. Road traffic accidents often lead to severe head injuries that can be effectively reduced by road measures. Moreover, there is an increased risk of head injuries among elderly patients and athletes [3,4]. Categorization of different types of head injury is challenging, as—mainly due to the diverse directions and magnitude of physical forces besides widely varying environmental conditions—the location, type, and extent of the induced pathoanatomical and pathophysiological sequelae may substantially differ case by case [5].

The estimated rate of post-TBI and TBI-related deaths worldwide is more than 1.5 million per year [6], mostly as a result of the lack of diagnostic abilities and/or life-saving brain surgeries [7].

The intensity and speed of the different forces acting on the head determine the extent of tissue damage. The brain essentially moves in the cerebrospinal fluid and various harmful mechanical forces, including shear and rotation, induce the displacement of the brain and corresponding tissue damage [8]. Based on pathoanatomical considerations, these lesions can be divided into focal and diffuse types. Focal damage usually occurs at the site of the impact or on the contralateral side of the brain. Meanwhile, focal lesions (epidural, subdural, intracerebral hemorrhages, and contusions) are more often associated with moderate or severe head injuries in most TBI cases and a mixture of focal and diffuse (edema, hypoxic–ischemic injury, microvascular injury, diffuse neuronal injury, and diffuse axonal injury) types of pathological lesions develop [9]. Diffuse pathological lesions—triggered by the shearing and rotational forces to the head—may occur in remote regions of the CNS (e.g., retina) compared to the direct impact site.

Based on the time course of the injury, we can speak of a primary injury that occurs at the time of the accident such as fractures and intrusions. On the other hand, secondary injury processes initiated at the moment of injury become clinically apparent hours or days after the initial impact (edema, ischemia, hypoxia, neuroinflammatory processes) and may develop even years or decades after the injury (such as chronic traumatic encephalopathy, post-traumatic stress disorder, dementia, or endocrine deficiencies), causing severe quality of life issues for the affected patients as well as a huge socio-economic burden [10]. Therefore, there is an unmet clinical need for efficient diagnostic (and prognostic) possibilities. The retina is not only one of our most important sensory organs, but it is also a part of our CNS, directly linked to the brain and located peripherally, thus providing easy access for diagnostic measures.

Studies show that characteristic retinal TBI symptoms include photophobia, double or blurred vision, visual paralysis, optic nerve disorders, and damaged or changed image processing [11]. TBI also includes visual dysfunction, which, according to current data, occurs in up to 90% of those affected by TBI [12]. Mapping these lesions and also milder effects on the retina may help to understand the pathophysiology of TBI and may be an indicative factor in screening for severe cases. Our work is important, pioneering research that seeks to expand our understanding of brain injuries and offers a potential diagnostic tool. Even when cellular and apoptotic lesions are present elsewhere in the brain, the retina can be a direct and most accessible indicator for TBI. However, at present, we lack the necessary information to connect direct retinal damage with TBI.

Microglia are the resident immune cells in the brain, including the retina. They are immunocompetent cells constantly monitoring their environment to interact with possible threats. Microglial activation in the retina is present in a cohort of disease phenotypes [13,14], hence it is important to understand how they contribute to the pathophysiology. In the central nervous system (CNS) their activation ranges on a continuum from neuroprotective to neurotoxic [15]. Their vast activation can contribute to further deterioration of the disease phenotype in the neuronal tissue via pro-inflammatory and phagocytosis mechanisms [13,16]. Caspase3 (Casp3) is a central effector for apoptotic cell death. It can be widely activated in TBI [17], and retinal diseases or detrimental conditions [18]. Microglial activation is highly dependent on caspase activation [19].

To the best of our knowledge, our study is the first to show how the retina is affected by sTBI and rmTBI. The latter is caused by multiple low-level impacts common among athletes (especially boxing and soccer) and is increasingly recognized as a major cause of neurological diseases [20]. Our results show rising numbers of activated microglial cells as well as activated Casp3 (act-Casp3)-positive cells in the retina in both traumatic models. Our data reinforce views that emphasize the severity of rmTBI and recommend attention similar to that given to sTBI.

Act-Casp3 is a common executor of different apoptotic pathways induced by various types of damage including ischemia, excitotoxicity, and radiation [21]. Microglia can show elevated levels of act-Casp3 without cellular death as they have a bypass mechanism to avoid apoptosis [19].

This suggests a different action in the course of the disease and points to new diagnostic procedures for TBI.

## 2. Results

**Figure 1:**
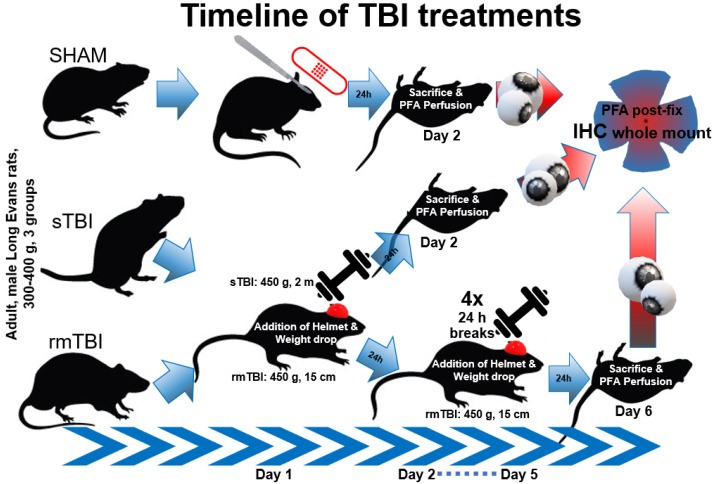
Timeline of TBI treatments. Different treatments in the experimental setup: SHAM-operated, sTBI (Marmarou, 450 g, single 2 m drop), rmTBI (Marmarou, 450 g, five 15 cm drops). All animals were sacrificed after 24 h and perfused with 4% PFA. Eyes were removed and post-fixed as eyecups until further IHC labeling (see Section 4.2).

Throughout the treatment, we used three animal model groups: SHAM-operated, sTBI, and rmTBI, and investigated differences between them. The latter were treated with either a high-impact (2 m), single drop of a weight or five consecutive low-height (15 cm) weight drops on a skull-fixed helmet to mimic severe and repetitive–mild injuries and to be able to differentiate between their effects. Twenty-four hours after the last treatment, the animals were sacrificed, and their retinas were fixed for further experiments (Figure 1).

### 2.1. Effects of TBI on Retinal Microglia

**Figure 2:**
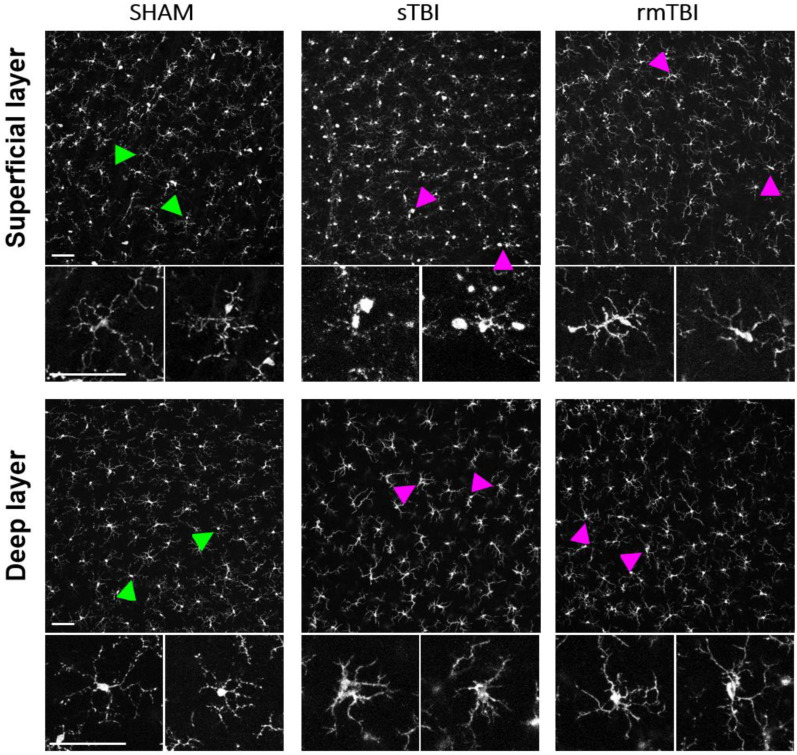
Microglial activation after sTBI and rmTBI in the retina. Microscope images show the activation of microglia in sTBI (Marmarou, 2 m) and rmTBI (3× Marmarou, 15 cm) in the rat retina. Green arrowheads show examples of non-activated microglia from SHAM retinas, magnified from the bottom to each arrowhead. Magenta arrowheads show microglia with activated morphology (described in the table in Section 4.4) in sTBI and rmTBI. Scale bar: 50 µm.

**Figure 3:**
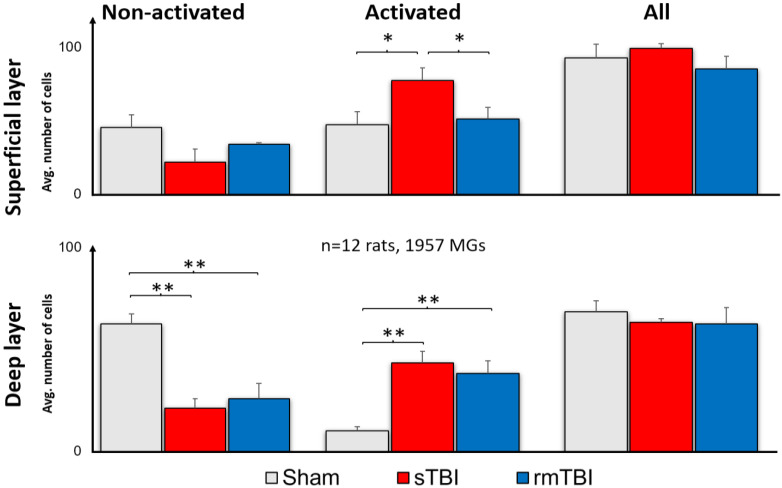
Microglial activation after sTBI and rmTBI in the retina. A significant increase in the number of activated microglia was observed after sTBI (Marmarou 2 m) and rmTBI (3× Marmarou 15 cm) in the deep layer of the rat retina. In the superficial layer, only sTBI resulted in microglial activation. There is no significant change in the total number of microglia. ANOVA, Gabriel post hoc, * *p* < 0.05, ** *p* < 0.01.

In both rmTBI and sTBI samples, extensive microglial activation could be observed on merged stacks of the superficial layer (SL) and the deep layer (DL) (Figure 2). This activation manifested in various morphological changes, including enlarged, disorganized soma, relative soma size increase, amoeboid and leaf-like structures on the dendritic endfeet, and aggregated occurrence of these activated microglial (MG) morphologies (details in Section 4.3 and the Table in Section 4.4). After counting and sorting microglial cells into activated/non-activated categories, according to the expressed morphological criteria (the Table in Section 4.4), we found that TBI resulted in a significant increase in the number of activated microglia in both the deep layer (mean = 43.3; Figure 3; Supplementary Table S1) and superficial MGs (mean = 64.35; Figure 3; Supplementary Table S1). However, this increase was observed only in sTBI among SL MGs, whereas only DL MGs were activated significantly in rmTBI (Figure 3).

### 2.2. Specific Caspase3 Activation due to Traumatic Brain Injury in the Superficial Layer of the Retina

Act-Casp3 is a reliable marker to summarize apoptotic cell death [21], therefore, we used it to show widespread apoptosis in the retina.

**Figure 4:**
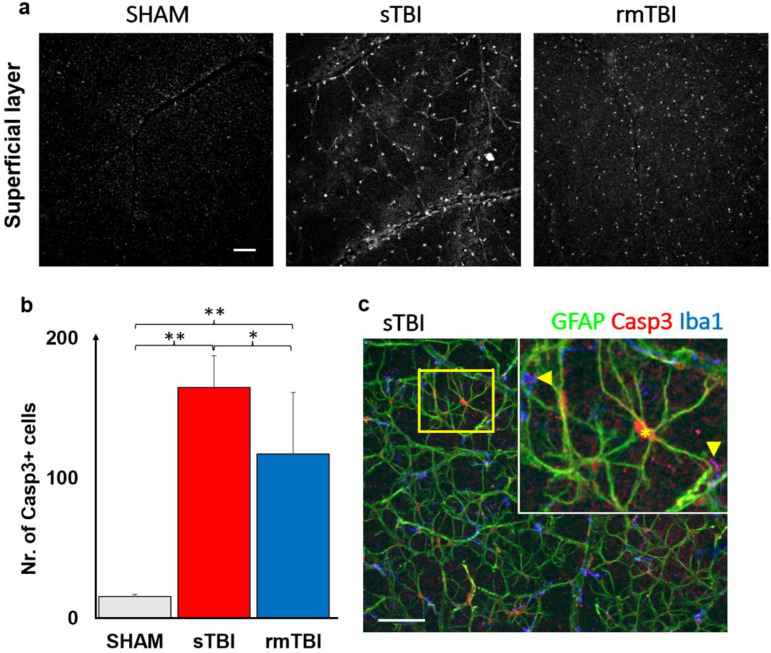
Casp3 activation in the SL of the retina. The figure shows an increase in the number of Casp3+ cells after sTBI and rmTBI compared to SHAM-operated animals (**a**). (**b**) A plot of the mean increase in Casp3+ cell numbers (one-way ANOVA, Gabriel post hoc, * *p* < 0.05, ** *p* < 0.01; see Supplementary Table S2 for details). (**c**) Following GFAP and Iba1 labeling, the Casp3+ cells were identified as astrocytes and microglia based on colocalizations with specific markers (microglia: arrows, astrocyte: *). Scale bar: 50 µm.

Act-Casp3 activation was analyzed first in the superficial layers of the retina, where Casp3 activation was already observed in confocal LSM images of both sTBI and rmTBI samples, which unfolded in two ways. On one hand, Casp3 was present in the nuclei or perinuclearly and in the cytosol of cells of a distinct cell population in the neurofilament layer, and on the other hand, it showed granular labeling in the ganglion cell layer (Figure 4c). We observed a large, 10.54× increase in the total number of act-Casp3 cells in sTBI, compared to SHAM (15.67 → 165.20), while a slightly smaller but still large 7.52× increase in activated cell number in rmTBI (15.67 → 117) (Figure 4b, Supplementary Table S1). We should note that in the case of rmTBI, the standard deviation of our sample was much larger, but still showed a significant prominent increment (Figure 4b, Supplementary Table S1).

The large number of act-Casp3 cells described was identified using several markers (non-correlated markings are not provided here). Finally, Casp3 activation in SL was found in colocalizations with the two markers. These markers were glial–fibrillar acidic protein (GFAP) and ionized calcium-binding adapter molecule 1 (Iba1), the signaling of which is mainly restricted to astrocytes and microglia, respectively, in the retina [22,23]. Using the related markers, we identified astrocytes and microglia in SL as the main source of Casp3+ labeling (Figure 4c).

Interestingly, the localization of the label differed between the two cell types. In GFAP+ astrocytes, the Casp3 signal was clearly restricted to the nucleus and its surrounding region, whereas the Casp3 localization of microglia was more limited to the protrusions. This could be observed in most of the cells in correlation with the Iba1 microglial marker (Figure 4c, arrows).

### 2.3. Loss of Axonal Connections due to Traumatic Brain Injury in the Neurofilament Layer of the Retina

**Figure 5:**
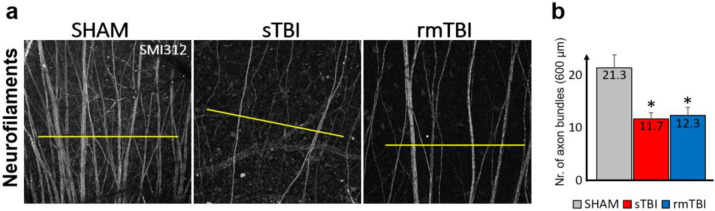
Change in SMI312 signal in the neurofilament layer in the retina after sTBI and rmTBI. (**a**) Images of the SMI312+ neurofilaments show a decline in both sTBI and rmTBI; 600 µm lines (yellow) were used to measure peaks of axon bundles after plot profile analysis, whereas the average number is shown by (**b**), indicating the crossed axon bundles (*n* = 3 each). Paired *t*-test, * *p* < 0.05.

Axonal injury and loss are the common concomitants in TBI, hence in addition to the obvious microglial activation and Casp3 activation we tested the SL of the rat retina with SMI312 (neurofilament H) labeling (prepared using SMI31 and 32 together) that showed a decline in the axonal numbers in the neurofilament layer (NFL) of the sTBI and rmTBI retinas in comparison to the SHAM (Figure 5a).

### 2.4. Caspase3 Is Activated due to Traumatic Brain Injury in the DL of the Retina

The DL is rich in cellular elements, including bipolar, amacrine, and horizontal cells in addition to the MGs found here, interacting with these cell types. Here, we tested if the two TBI models have a different effect on these cells.

**Figure 6:**
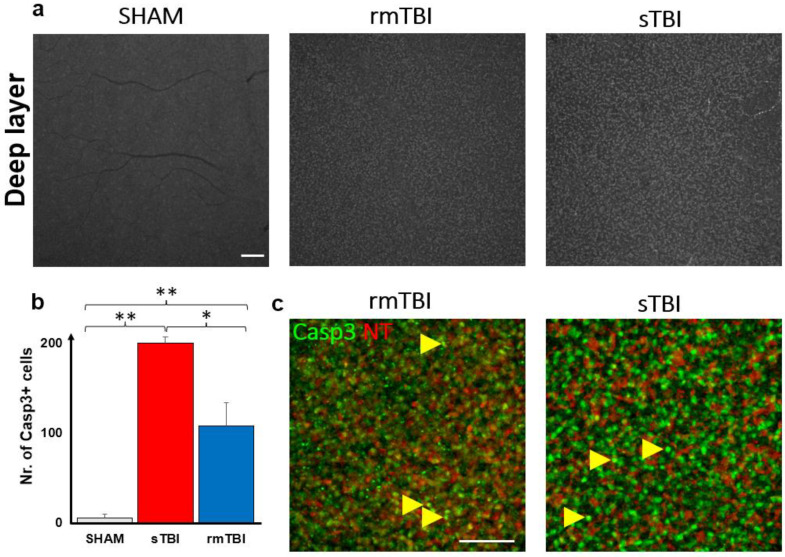
Casp3 activation in the deep layers of the retina. The figure shows an increase in the number of Casp3+ cells (**a**: gray, **c**: green) after sTBI and rmTBI compared to SHAM-operated animals (**a**). (**b**) A plot of the mean increase in Casp3+ cell numbers (one-way ANOVA, Gabriel post hoc, * *p* < 0.05, ** *p* < 0.01; details in Supplementary Table S3). NeuroTrace-640 (NT, red) labeling helped to identify these cells as bipolar cells and possibly Müller cells in the magnified images ((**c**), shown with yellow arrows, not labeled with NT). Scale bar: 50 µm.

Subsequent analyses of the Casp3 labeling in the DL unfolded in two ways. A massive (21.5- and 39.9-fold) increase in Casp3 activation was obvious after counting the individual cells with the help of NT labeling (Figure 6b,c, Supplementary Table S3) in both the rm- and sTBI samples.

After closer examination, we identified the Casp3+ cells mainly as bipolar cells based on their soma diameters (mainly from 4–7 µm) [24] and clear colabeling with NT. A second cohort of cells were not NT labeled with bright Casp3 labeling. The soma diameters of these latter cells were larger compared to bipolar cells and the somas were located distally from the innermost sublamina of the INL where amacrine cells could be observed (based on the strong NT labels and the relatively large, 7–14 µm somata [25]). Therefore, this second cohort of non-NT-labeled Casp3+ cells were identified as Müller cells.

## 3. Discussion

Retinal ganglion cells (RGCs) actively project their axons to over 40 subcortical brain regions [26,27]. RGC axons are openly exposed to the effects of different injuries (sTBI or rmTBI) and the retina can actively intercept signals from the brain tissue, projected by axonal connections and indicated by the cells of the retina [28,29], including MGs as primary immune cells.

### 3.1. Microglial Activation due to Traumatic Brain Injury

Based on our results, microglial activation clearly occurred in both the sTBI and rmTBI models. This suggests that the assumption from previous research about only 40% of TBI patients having a negative retinal impact appears to be highly underestimated [12,29]. Surprisingly, although we observed a high level of activation in sTBI, the data measured in rmTBI were close to those measured in sTBI (Figure 2 and Figure 3), which may be alarming in cases where someone (e.g., in sports such as football, boxing) is continuously exposed to minor cranial injuries [30]. SMI312 labels ganglion cells, including their axons in control retinas. Axons in the NFL show a decline in the SMI312 labeling after both types of treatment (Figure 5), similarly to other related studies working with synaptophysin [29]. Compared to that, the microglia show differential SL/DL activation in sTBI and rmTBI. Our results show no significant microglial activation in the SL after rmTBI, only after sTBI, whereas in the deep layer microglia are similarly activated in the DL of the retina (Figure 3). The possible explanation for this could be the different effect that is mediated by rmTBI but further studies should be carried out to unravel the background of this difference in activation.

It is important to mention that microglial cells can have different morphologies in the retina following the spatial organization patterns of other cells but the rat retina does not have a fovea (nor a pronounced central zone or visual stripe) as in many other animals, that may result in microglial differences [31], therefore, in our study we did not analyze these topological variations. However, to avoid any baseline difference our measurements were made from a mid-central region of the rat retina.

Microglial cells continuously scan their environment, and their activation state faithfully reflects the state of the retina and changes that are taking place in the tissue, hard-wired to diverse parts of the brain. The activation status of microglia may be different (M0, -1, -2) and treatment options may vary accordingly. Many drugs (e.g., CB2 inverse agonists) can affect the activation state of microglia and can reverse the noxious inflammatory M1 phase to the reparative M2 [32]. The differential activation of microglia can be of further interest for the right treatment of TBI-induced disease.

However, in addition to the choice of treatment options, activation of microglial cells could be used as a biomarker of TBI in the future, as these cells may be visualized by a combination of different staining procedures and fluorescent coherent optical tomography (fOCT) methods [33]. As not all lesions can be traced with current imaging methods, the development of new in vivo procedures is needed. As shown before, vital stains can help in aiming for different cell types in the retina, such as NeuroTrace in pericytes [34]. However, no real breakthrough has yet been made in the development of imaging methods or microglia-specific live stains in the retina or the brain. Only one technique, adaptive optics-based imaging, could be able to breach this limitation at present, where we can see individual cell morphologies in the living eye [35].

Activated microglia can release a number of factors (e.g., tumor necrosis factor α, interleukin-1β) that can induce inflammatory and degenerative processes in the retina [36]. Unfortunately, we are not able provide any labeling on these at this time, only Casp3, however, in the future labeling these factors could indicate microglial state since the neuroprotective effect of microglia is also well known, which can be a major help in preserving vision [37]. The detection and further examination of these factors represent great advances in determining the exact role of microglia in TBI or measuring their activation state.

### 3.2. Caspase3 Activation, Cell Death Marker

Caspases play a role in both apoptosis and inflammatory processes [19,38,39]. Animal studies have demonstrated activation of caspases (3, 7, 9, 12) in TBI in the brain [40,41], where Casp3 activation is a key, cumulative marker of cell death since it signals the onset of both the intrinsic and extrinsic pathways of apoptosis. Studies suggest dimerization as part of activation that binds inactive monomers together [42,43]. We chose Casp3, together with other markers, to summarize apoptotic processes. One of the main markers that we used as a global cell marker is NT640. This fluoro-Nissl stain labels neuronal cells and vascular cells (mainly pericytes) in the fixed retina and is also capable of labeling living cells if used in the right concentration [34], therefore it was the perfect candidate to pair with Casp3 to see how the neurons and some other cells are affected. Other markers such as GFAP only show astrocytes and strongly affected Müller cell endfeet [44]. According to our results, the SL clearly indicates Casp3 activation in GFAP+ cells, which we identified as astrocytes. Astrocyte activation also occurs in high intraocular pressure (IOP) conditions, such as glaucoma [45].

Further markers can subserve to identify apoptotic cell types. However, in the DL of the retina, it is difficult to identify distinct cell types expressing the Casp3+ signal due to the greater number of cells, the many cell types, the relatively small soma sizes as well as the lack of cell type-specific markers (e.g., amacrine, bipolar subtypes). We, however, tried to underline the fact that bipolar and amacrine cells could be easily differentiated based on their soma size. Previous Prox1 labeling in the INL to determine cell size differences showed ~35% amacrine vs. bipolar size difference (amacrine diameters for cat: 9.65 ± 0.29 μm, *n* = 9; rat: 8.27 ± 0.14 μm, *n* = 15; mouse: 9.33 ± 0.19 μm, *n* = 21, vs. bipolar: cat: 6.09 ± 0.09 μm, *n* = 238, *p* < 0.001, rat: 5.33 ± 0.14 μm, *n* = 344, *p* < 0.001, mouse: 6.30 ± 0.11 μm, *n* = 379, *p* < 0.001 in *t*-test against horizontal cell profiles, in [46]). On the other hand, various cell types involved can be more easily identified with cell-type-specific markers labeling the SL cells with fine morphological detail, therefore we used SMI312 and GFAP. By quantifying the labeling, and also adding SMI312 and GFAP, resulting in clear cellular morphology, we were able to identify the activated cells. In the GFAP+ population, the cells’ nuclei were colabeled with Casp3, and we were able to visualize subcellular granular Casp3+ labeling colocalized with Iba1. As GFAP labels the astrocytes, and Iba1 the MGs, in the retina [18,47], we identified them as the most affected population in both TBI models. However, the differential labels in the two cell populations may indicate that Casp3 might have different effects on them.

The neuronal marker SMI312, in some cells, coexpressed with Casp3, indicates the involvement of RGCs, however, we did not encounter extensive labeling of neurons. The axonal degeneration is obvious by the lack of SMI312+ axonal labeling in both sTBI and rmTBI. This result thus coincided with neuronal survival in similar works by other research groups [48]. However, it may be that more neuronal Casp3 activation could have been observed with longer survival time, giving more time for apoptotic processes.

The extent of diffuse axonal injury (DAI) in sTBI and rmTBI may come from other brain areas. This type of injury first arises in the axonal head and by disorganization of the cytoskeleton and transport mechanisms, which in turn deteriorates the axon and the soma of the affected cell through a Ca2^+^ and calpain-induced process [49]. Even now, it is still a matter of debate if the surviving GCs are able to maintain normal visual function in the long term [48,49,50], thus further studies are required in the future on this subject.

In addition to neuronal colocalization, the use of GFAP was evident in the retina, as activation of astrocytes by TBI could also be expected. The results we obtained show that astrocytes began large-scale mass activation of Casp3, providing a clear sign of apoptosis. The astrocyte network is responsible for the health of retinal neurons by maintaining homeostasis and involvement in the blood–retinal barrier [51].

We observed Casp3 activation of microglia in both rm- and sTBI. However, it is important to note that activation of Casp3 in microglia does not necessarily imply a commitment toward cell death, as these cells have a bypass mechanism to avoid cell death. In microglial cells, various inflammatory factors induce the activation of Casp8 and then Casp3. Active Casp3, in turn, promotes the activation of microglial inflammatory pathways through a protein kinase Cδ-dependent pathway without induction of cell death [19]. Activation of Casp3 occurs as a two-step process in which the zymogen is first cleaved by upstream caspases, such as Casp8, to form an intermediate but active cytoplasmic p19/p12 complex. An autocatalytic process then creates the fully mature form of the enzyme p17/p12, which is then transferred to the nucleus. Induction of the cellular inhibitor of apoptosis protein 2 (cIAP2) upon microglial activation prevents the conversion of the p19 subunit of Casp3 to the p17 subunit, which is responsible for the cessation of Casp3 activity. By reversing the cIAP2-dependent process, the repressive effects are exerted, reactivate the inflammatory function of microglial cells, and may eventually promote their death [52]. We can assume that somehow Casp3 accumulation might act as a key for phenotype change.

Considering this, however, in our case we do not expect extensive cell death in many Casp3+ microglia. Instead, our results, as such, are further evidence of the activation of microglial cells beyond the morphological features. These microglia do not necessarily induce cell death and phagocytes but may be involved in tissue protection [52] for cells in which Casp3 activation is not associated with the process. Therefore, Casp3 activation can be an additional marker for TBI.

### 3.3. Fate of Different Cell Types under the Influence of TBI

As mentioned in combination with Casp3, other cell-specific markers can be identified. However, with the markers we used, we could only draw the following conclusions. Only a limited number of markers could be used in the experiments. Unfortunately, we could not coadminister SMI312 with Casp3 in the entire sample set due to the lack of available secondary channels in IHC. However, no cell-specific Casp3 activation was examined with further specific markers, but damage to other cell types could not be completely ruled out. This can be observed through further experiments in the future.

GFAP may be expressed in Müller cell endings [45] in some cases due to severe damage, however, no Müller cell endings were observed with the GFAP label, morphologically, based on our experiments. Since no Müller cell-specific marker was used (e.g., glutamine synthetase, Sox9, RLBP9; [53]), their involvement cannot be completely ruled out based on our study.

SMI312 labels both the non-phosphorylated and phosphorylated neurofilament H (NF-H) and is therefore specific for RGCs [54]. This marker potentially labels living cells, hence the intermediate filaments decompose in apoptosis [55]. NF-H can also be detected in serum after the onset of DAI, peaking at 12–48 h after injury [49]. NF-H is considered the most convenient marker of DAI diagnosis and, together with MG imaging, this might be the next step for a more accurate diagnosis of TBI pathology in the future.

In conclusion, we showed that both sTBI and rmTBI comparably had a detrimental effect on the retina with the exception of DL activation, where rmTBI had less effect. We showed that act-Casp3 is elevated in numerous cells, identifying astrocytes and microglia as major contributors. In the DL, act-Casp3 is present in bipolar and Müller cells. We also identified a progressive decline in SMI312+ axonal bundles suggesting an interference with further cellular connections. Our results highlight the importance of retinal involvement in TBI and suggest that monitoring the retina’s health status could be utilized as a future biomarker tool for diagnostic purposes, able to detect the fine changes shown here.

## 4. Materials and Methods

### 4.1. Animals and Preparation

Animal handling, housing, and experimental procedures were reviewed and approved by the ethical committee of the University of Pécs (BA02/2000-69/2017). Adult, male Long Evans rats (*n* = 12, Charles River Laboratory, Göttingen, Germany) weighing 300–400 g were used in the experiment. All animals were treated in accordance with the ARVO Statement for the Use of Animals in Ophthalmic and Vision Research. All efforts were made to minimize pain and discomfort during the experiments.

TBI injuries were performed identically to Tadepalli et al. [56]. The methodological background had been established and the outcomes of both sTBI and rmTBI were proven here to similarly affect the brain. Briefly, to induce experimental TBI, we used an impact acceleration weight-drop model, originally published by Marmarou and Foda [57,58,59,60]. The steps of the surgical protocol are explained briefly as follows. Anesthesia was induced in an induction box with 5% isoflurane (Baxter, Budapest, Hungary) in a 70:30 N2:O2 gas mixture. Once the anesthesia stabilized, we fixed the animal’s head in a stereotaxic frame. From this point, the anesthesia was carried out with 2% isoflurane in the same gas mixture. After removing the hair from the animal’s scalp, we made a midline incision and removed the periosteum associated with the top of the skull. Halfway between the exposed bregma and lambda sutures, we fixed a stainless-steel disc, the so-called “helmet”, directly to the bone with cyanoacrylate glue. Then, we laid the animal on a foam bed in a prone position. The helmet was positioned centrally under s weight-leading plexiglass tube. Experimental diffuse TBI was induced by dropping the weight from the height corresponding to the desired severity level. After TBI induction, the helmet was removed, and the surgical area was cleaned and disinfected. The wound was sutured, and the animal was returned to its cage to recover. Through the surgical procedure, the physiologic parameters of the animals were monitored by a pulse oximeter (MouseOx Plus, Starr Life Corp., Oakmont, PA, USA). Body temperature was monitored by the Homothermic Monitoring System (Harvard Apparatus, Holliston, MA, USA) and maintained with a heating pad on the same device at 37 °C.

To investigate the acute pathological effects of experimental diffuse TBI on the retina, we divided the animals into 3 groups (*n* = 4 in each group). In the first, single severe TBI (sTBI) group, we induced the injury with a 450 g weight from a 2 m height. In the second, repetitive mild TBI (rmTBI), group we used the same weight from a 15 cm height 5 times with 24 h intervals. Finally, to eliminate the possible effects of anesthesia/environmental conditions, our third group (SHAM) did not receive weight-drop treatment, only anesthesia, and the surgical protocol of fixing the helmet on the skull. This experimental design allowed us to investigate pathological alterations between injuries of different degrees of severity and frequency.

Following the treatments, the animals were sacrificed after 24 h. Control and TBI rats were perfused transcardially with 4% PFA (4% paraformaldehyde in PBS: 137 mM NaCl; 2.7 mM KCl; 10 mM Na_2_HPO_4_·7H_2_O, pH 7.4), and their eyes were immediately removed. The eyes were dissected in PBS by removing the cornea and lens. The resulting eyecups were additionally fixed in 4% PFA at room temperature for 15 min for better sample retention. After washing them three times for 10 min in PBS, the retinas were dissected from the eyecups, or the eyecups were kept for up to 3 weeks in 0.05% Na-azide in PBS at 4 °C until processed (for details, see Figure 1).

### 4.2. Immunohistochemistry

Flat-mounted retinas were blocked in 100 μL of CTA (5% Chemiblocker, 0.5% TritonX-100, 0.05% Na-azide in PBS) overnight, room temp., humidified. After blocking, the retinas were treated with the primary antibodies (1000× + 1000× mouse SMI31 + SMI32 = SMI312, NE1022/NE1023—Calbiochem; 1000× rabbit Caspase3, AF835—NovusBio; 2000× guinea pig Iba1, 234,004—SySy), diluted in CTA, for 48 h, room temperature (RT). Retinas were incubated for 48 h at RT. After subsequent washing in PBS, three times, secondary antibodies were applied: 500× anti-rabbit Cy3 (715-165-150—Jackson), 1000× anti-mouse A488 (A11017—Invitrogen), and 1000× anti-mouse A647 (A21237—Invitrogen) or anti-guinea pig A647 (A21450—Invitrogen) in CTA, and incubated overnight at room temperature (see Table 1 for antibodies). After washing three times for 10 min in PBS, they were coverslipped with Vectashield (Vector Laboratories, Peterborough, UK) using coverslip nr. 1 (protocol based on and previously validated in [18,61]).

### 4.3. Microscopy

Retinas were inspected using a Zeiss LSM 710 confocal laser scanning microscope (Plan Apochromat 10×, 20×, and 63× objectives (NA: 0.45, 0.8, 1.4, Carl Zeiss Inc., Jena, Germany)) with normalized laser power and filter settings making 1.5 and 0.5 μm thin optical sections (further details in Balogh, 2021 [18]).

### 4.4. Measurement of Microglial and Casp3 Activation

All measurements were performed using FIJI (NIH, Bethesda, MD, USA, [62]). First, we performed two z-merges from the 5–5 optical stacks (3.75 µm) for the superficial and deep regions of MGs using only scans from mid-central retinal regions. The microglia were separated from the other signals based on their expression of ionized calcium-binding adapter molecule 1 (Iba1). Cells were manually grouped one by one according to their morphologies into activated and non-activated, using the “Cell-counter” plugin in FIJI, according to the morphological classifications of Lawson and colleagues and others [63,64,65]. Only cells with the whole visible area were included, we omitted the ones on the edges. In order to objectively verify the choices of the trained observers, reconstructions of the dendritic arbor of every fifth recorded MG were created using the Simple Neurite Tracer plugin [66] of FIJI. Subsequently, Sholl analysis was performed (1 μm radius) and the following relevant parameters were measured (Green and colleagues [67]): (1) peak values, (2) total number of dendrites, (3) total length of dendrites, (4) total number of branches, (5) path orders, (6) convex hull size, elongation, and roundness, utilizing the same plugin. Finally, the maximum cross-sectional area of the soma and the dendritic field was measured, from which a ratio was calculated (Supplementary Figure S1). These parameters were used to compare the cells classified into resting and reactive morphologies by the trained observer, confirming their choices, and they were also used to determine the effect of the TBI treatment on the MGs in the retina, an example of which can be seen in Supplementary Figure S1f–h.

We divided superficial (SL) and deep layers (DL) in the z-stacks for MGs in the 20× images following the layers of blood vessels, overlapping NFL+GCL for SL, and INL to ONL for DL. A “z-merge” was carried out from 5 consecutive optical sections. During microglial activation, counting and classifying cells with different morphologies (*n* = 1957 cells in 12 retinas) were performed one by one using the “Cell-counter” plugin in FIJI. Differential morphological characteristics listed in Table 2 (based on the work of Davis and his colleagues [68]) were considered during selection. To preserve objectivity, the names of sample images were randomized.

To examine Casp3 activation in the SL and DL, we used the same z-merges, and the activated cells were separated from the background using other signals (GFAP, SMI312) together with the act-Casp3 signal to define retinal layer borders better and possible cell types affected.

### 4.5. Statistical Analysis

Data were curated in MS Excel. One-way ANOVA analyses were performed using Origin18 (Origin, Version 2018b, OriginLab Corporation, Northampton, MA, USA) and JASP (JASP Team (2022) (Version 0.16.3)). Normal distribution was previously confirmed through statistical analysis.

## Figures and Tables

**Table 1:** Antibodies.

Primary Antibodies				Secondary Antibodies, Dyes			
Name	Dilution	Source	Code	Name	Dilution	Source	Code
ms-SMI312	1:1000	Calbiochem	NE1022/NE1023	anti-ms-Alexa488	1:1000	Invitrogen	A11017
gp-Iba1	1:2000	SySy	234004	anti-ms-Alexa647	1:1000	Invitrogen	A21237
rb-Caspase-3	1:1000	NovusBio	AF835	anti-gp-Alexa647	1:1000	Invitrogen	A21450
				anti-rb-Cy3	1:500	Jackson	715-165-150

**Table 2:** Morphological differences between non-activated and activated microglia.

Non-Activated (Naïve) Morphology	Activated Morphology
Small, round soma	Enlarged, disorganized soma
Low soma to surroundings ratio	Soma to surroundings ratio increases
No amoeboid or leaf-like appendages are found	Amoeboid and leaf-like structures
Sporadic occurrence of act-MGs	Aggregate occurrence of act-MGs

## Data Availability

Data are available upon request.

## Supplementary Materials

The supporting information can be downloaded at: https://www.mdpi.com/article/10.3390/ijms24054451/s1.

## References

[1] Orff H.J., Ayalon L., Drummond S.P.A. (2009). Traumatic Brain Injury and Sleep Disturbance: A Review of Current Research. J. Head Trauma Rehabil..

[2] Jennekens N., de Casterlé B.D., Dobbels F. (2010). A Systematic Review of Care Needs of People with Traumatic Brain Injury (TBI) on a Cognitive, Emotional and Behavioural Level. J. Clin. Nurs..

[3] Harvey L.A., Close J.C.T. (2012). Traumatic Brain Injury in Older Adults: Characteristics, Causes and Consequences. Injury.

[4] Ling H., Hardy J., Zetterberg H. (2015). Neurological Consequences of Traumatic Brain Injuries in Sports. Mol. Cell. Neurosci..

[5] Hamel R.N., Smoliga J.M. (2019). Physical Activity Intolerance and Cardiorespiratory Dysfunction in Patients with Moderate-to-Severe Traumatic Brain Injury. Sports Med..

[6] de Souza R.L., Thais M.E., Cavallazzi G., Diaz A.P., Schwarzbold M.L., Nau A.L., Rodrigues G.M., Souza D.S., Hohl A., Walz R. (2015). Side of Pupillary Mydriasis Predicts the Cognitive Prognosis in Patients with Severe Traumatic Brain Injury. Acta Anaesthesiol. Scand..

[7] van Dijck J.T.J.M., Bartels R.H.M.A., Lavrijsen J.C.M., Ribbers G.M., Kompanje E.J.O., Peul W.C. (2019). The Patient with Severe Traumatic Brain Injury: Clinical Decision-Making: The First 60 min and Beyond. Curr. Opin. Crit. Care.

[8] Zhou Z., Li X., Kleiven S. (2020). Biomechanics of Periventricular Injury. J. Neurotrauma.

[9] Mckee A.C., Daneshvar D.H. (2015). The Neuropathology of Traumatic Brain Injury. Handb. Clin. Neurol..

[10] Capizzi A., Woo J., Verduzco-Gutierrez M. (2020). Traumatic Brain Injury: An Overview of Epidemiology, Pathophysiology, and Medical Management. Med. Clin. N. Am..

[11] Das M., Tang X., Mohapatra S.S., Mohapatra S. (2019). Vision Impairment after Traumatic Brain Injury: Present Knowledge and Future Directions. Rev. Neurosci..

[12] Ciuffreda K.J., Kapoor N., Rutner D., Suchoff I.B., Han M.E., Craig S. (2007). Occurrence of Oculomotor Dysfunctions in Acquired Brain Injury: A Retrospective Analysis. Optometry.

[13] Guo L., Choi S., Bikkannavar P., Cordeiro M.F. (2022). Microglia: Key Players in Retinal Ageing and Neurodegeneration. Front. Cell. Neurosci..

[14] Rashid K., Akhtar-Schaefer I., Langmann T. (2019). Microglia in Retinal Degeneration. Front. Immunol..

[15] Hellwig S., Heinrich A., Biber K. (2013). The Brain’s Best Friend: Microglial Neurotoxicity Revisited. Front. Cell. Neurosci..

[16] Shi Y., Manis M., Long J., Wang K., Sullivan P.M., Serrano J.R., Hoyle R., Holtzman D.M. (2019). Microglia Drive APOE-Dependent Neurodegeneration in a Tauopathy Mouse Model. J. Exp. Med..

[17] Clark R.S.B., Kochanek P.M., Watkins S.C., Chen M., Dixon C.E., Seidberg N.A., Melick J., Loeffert J.E., Nathaniel P.D., Jin K.L. (2000). Caspase-3 Mediated Neuronal Death After Traumatic Brain Injury in Rats. J. Neurochem..

[18] Balogh B., Szarka G., Tengölics Á.J., Hoffmann G., Völgyi B., Kovács-öller T. (2021). Led-induced Microglial Activation and Rise in Caspase3 Suggest a Reorganization in the Retina. Int. J. Mol. Sci..

[19] Burguillos M.A., Deierborg T., Kavanagh E., Persson A., Hajji N., Garcia-Quintanilla A., Cano J., Brundin P., Englund E., Venero J.L. (2011). Caspase Signalling Controls Microglia Activation and Neurotoxicity. Nature.

[20] Eyolfson E., Khan A., Mychasiuk R., Lohman A.W. (2020). Microglia Dynamics in Adolescent Traumatic Brain Injury. J. Neuroinflamm..

[21] Mcilwain D.R., Berger T., Mak T.W., Baehrecke E.H., Green D.R., Kornbluth S., Salvesen G.S. (2013). Caspase Functions in Cell Death and Disease. Cold Spring Harb. Perspect. Biol..

[22] Bruera M.G., Benedetto M.M., Guido M.E., Degano A.L., Contin M.A. (2022). Glial Cell Response to Constant Low Light Exposure in Rat Retina. Vis. Neurosci..

[23] Zhang J., Wu G., Ishimoto S.-I., Pararajasegaram G., Rao N.A. (1997). Expression of Major Histocompatibility Complex Molecules in Rodent Retina. Immunohistochemical Study. Investig. Ophthalmol. Vis. Sci..

[24] Tsukamoto Y., Omi N. (2017). Classification of Mouse Retinal Bipolar Cells: Type-Specific Connectivity with Special Reference to Rod-Driven AII Amacrine Pathways. Front. Neuroanat..

[25] Liu J.H., Singh J.B., Veruki M.L., Hartveit E. (2021). Morphological Properties of the Axon Initial Segment-like Process of AII Amacrine Cells in the Rat Retina. J. Comp. Neurol..

[26] Seabrook T.A., Burbridge T.J., Crair M.C., Huberman A.D. (2017). Architecture, Function, and Assembly of the Mouse Visual System. Annu. Rev. Neurosci..

[27] Lukas T.J., Wang A.L., Yuan M., Neufeld A.H. (2009). Early Cellular Signaling Responses to Axonal Injury. Cell Commun. Signal..

[28] Mannix R., Monuteaux M.C., Schutzman S.A., Meehan W.P., Nigrovic L.E., Neuman M.I. (2013). Isolated Skull Fractures: Trends in Management in US Pediatric Emergency Departments. Ann. Emerg. Med..

[29] Rasiah P.K., Geier B., Jha K.A., Gangaraju R. (2021). Visual Deficits after Traumatic Brain Injury. Histol. Histopathol..

[30] Fehily B., Fitzgerald M. (2017). Repeated Mild Traumatic Brain Injury. Cell Transplant..

[31] Singaravelu J., Zhao L., Fariss R.N., Nork T.M., Wong W.T. (2017). Microglia in the Primate Macula: Specializations in Microglial Distribution and Morphology with Retinal Position and with Aging. Brain Struct. Funct..

[32] Honig M.G., del Mar N.A., Henderson D.L., O’Neal D., Doty J.B., Cox R., Li C., Perry A.M., Moore B.M., Reiner A. (2021). Raloxifene Modulates Microglia and Rescues Visual Deficits and Pathology After Impact Traumatic Brain Injury. Front. Neurosci..

[33] Childs C., Barker L.A., Gage A., Loosemore M. (2018). Investigating Possible Retinal Biomarkers of Head Trauma in Olympic Boxers Using Optical Coherence Tomography. Eye Brain.

[34] Kovacs-Oller T., Ivanova E., Bianchimano P., Sagdullaev B.T. (2020). The Pericyte Connectome: Spatial Precision of Neurovascular Coupling Is Driven by Selective Connectivity Maps of Pericytes and Endothelial Cells and Is Disrupted in Diabetes. Cell Discov..

[35] Hammer D.X., Agrawal A., Villanueva R., Saeedi O., Liu Z. (2020). Label-Free Adaptive Optics Imaging of Human Retinal Macrophage Distribution and Dynamics. Proc. Natl. Acad. Sci. USA.

[36] Hernandez-Ontiveros D.G., Tajiri N., Acosta S., Giunta B., Tan J., Borlongan C.V. (2013). Microglia Activation as a Biomarker for Traumatic Brain Injury. Front. Neurol..

[37] Jin N., Gao L., Fan X., Xu H. (2016). Friend or Foe? Resident Microglia vs Bone Marrow-Derived Microglia and Their Roles in the Retinal Degeneration. Mol. Neurobiol..

[38] Kumar S. (1999). Mechanisms Mediating Caspase Activation in Cell Death. Cell Death Differ..

[39] Hengartner M.O. (2000). The Biochemistry of Apoptosis. Nature.

[40] Knoblach S.M., Nikolaeva M., Huang X., Fan L., Krajewski S., Reed J.C., Faden A.I. (2004). Multiple Caspases Are Activated after Traumatic Brain Injury: Evidence for Involvement in Functional Outcome. J. Neurotrauma.

[41] Glushakov A.O., Glushakova O.Y., Korol T.Y., Acosta S.A., Borlongan C.V., Valadka A.B., Hayes R.L., Glushakov A.V. (2018). Chronic Upregulation of Cleaved-Caspase-3 Associated with Chronic Myelin Pathology and Microvascular Reorganization in the Thalamus after Traumatic Brain Injury in Rats. Int. J. Mol. Sci..

[42] Boatright K.M., Salvesen G.S. (2003). Caspase Activation. Biochem. Soc. Symp..

[43] Boatright K.M., Salvesen G.S. (2003). Mechanisms of Caspase Activation. Curr. Opin. Cell Biol..

[44] Liu Y.X., Sun H., Guo W.Y. (2022). Astrocyte Polarization in Glaucoma: A New Opportunity. Neural. Regen. Res..

[45] Szabo E., Patko E., Vaczy A., Molitor D., Csutak A., Toth G., Reglodi D., Atlasz T. (2021). Retinoprotective Effects of PACAP Eye Drops in Microbead-Induced Glaucoma Model in Rats. Int. J. Mol. Sci..

[46] Fusz K., Kovács-öller T., Kóbor P., Szabó-Meleg E., Völgyi B., Buzás P., Telkes I. (2021). Regional Variation of Gap Junctional Connections in the Mammalian Inner Retina. Cells.

[47] Cardona S.M., Mendiola A.S., Yang Y.C., Adkins S.L., Torres V., Cardona A.E. (2015). Disruption of Fractalkine Signaling Leads to Microglial Activation and Neuronal Damage in the Diabetic Retina. ASN Neuro..

[48] Wang J., Fox M.A., Povlishock J.T. (2013). Diffuse Traumatic Axonal Injury in the Optic Nerve Does Not Elicit Retinal Ganglion Cell Loss. J. Neuropathol. Exp. Neurol..

[49] Ma J., Zhang K., Wang Z., Chen G. (2016). Progress of Research on Diffuse Axonal Injury after Traumatic Brain Injury. Neural Plast..

[50] Klimo K.R., Stern-Green E.A., Shelton E., Day E., Jordan L., Robich M., Racine J., McDaniel C.E., VanNasdale D.A., Yuhas P.T. (2022). Structure and Function of Retinal Ganglion Cells in Subjects with a History of Repeated Traumatic Brain Injury. Front. Neurol..

[51] Vecino E., Rodriguez F.D., Ruzafa N., Pereiro X., Sharma S.C. (2016). Glia–Neuron Interactions in the Mammalian Retina. Prog. Retin. Eye Res..

[52] Kavanagh E., Rodhe J., Burguillos M.A., Venero J.L., Joseph B. (2014). Regulation of Caspase-3 Processing by CIAP2 Controls the Switch between pro-Inflammatory Activation and Cell Death in Microglia. Cell Death Dis..

[53] Pellissier L.P., Hoek R.M., Vos R.M., Aartsen W.M., Klimczak R.R., Hoyng S.A., Flannery J.G., Wijnholds J. (2014). Specific Tools for Targeting and Expression in Müller Glial Cells. Mol. Ther. Methods Clin. Dev..

[54] Zhang C., Guo Y., Slater B.J., Miller N.R., Bernstein S.L. (2010). Axonal Degeneration, Regeneration and Ganglion Cell Death in a Rodent Model of Anterior Ischemic Optic Neuropathy (RAION). Exp. Eye Res..

[55] Tan H., Li X., Huang K., Luo M., Wang L. (2022). Morphological and Distributional Properties of SMI-32 Immunoreactive Ganglion Cells in the Rat Retina. J. Comp. Neurol..

[56] Tadepalli S.A., Bali Z.K., Bruszt N., Nagy L.V., Amrein K., Fazekas B., Büki A., Czeiter E., Hernádi I. (2020). Long-Term Cognitive Impairment without Diffuse Axonal Injury Following Repetitive Mild Traumatic Brain Injury in Rats. Behav. Brain Res..

[57] Marmarou A., Abd-Elfattah Foda M.A., van den Brink W., Campbell J., Kita H., Demetriadou K. (1994). A New Model of Diffuse Brain Injury in Rats: Part I: Pathophysiology and Biomechanics. J. Neurosurg..

[58] Chakraborty N., Hammamieh R., Gautam A., Miller S.A., Condlin M.L., Jett M., Scrimgeour A.G. (2021). TBI Weight-Drop Model with Variable Impact Heights Differentially Perturbs Hippocampus-Cerebellum Specific Transcriptomic Profile. Exp. Neurol..

[59] Johnson V.E., Meaney D.F., Cullen D.K., Smith D.H. (2015). Animal Models of Traumatic Brain Injury. Handb. Clin. Neurol..

[60] Büchele F., Morawska M.M., Schreglmann S.R., Penner M., Muser M., Baumann C.R., Noain D. (2016). Novel Rat Model of Weight Drop-Induced Closed Diffuse Traumatic Brain Injury Compatible with Electrophysiological Recordings of Vigilance States. J. Neurotrauma.

[61] Kovács-Öller T., Szarka G., Tengölics Á.J., Ganczer A., Balogh B., Szabó-Meleg E., Nyitrai M., Völgyi B. (2020). Spatial Expression Pattern of the Major Ca2+-Buffer Proteins in Mouse Retinal Ganglion Cells. Cells.

[62] Schindelin J., Arganda-Carreras I., Frise E., Kaynig V., Longair M., Pietzsch T., Preibisch S., Rueden C., Saalfeld S., Schmid B. (2012). Fiji: An Open-Source Platform for Biological-Image Analysis. Nat. Methods.

[63] Masuda T., Sankowski R., Staszewski O., Prinz M. (2020). Microglia Heterogeneity in the Single-Cell Era. Cell Rep..

[64] Lawson L.J., Perry V.H., Dri P., Gordon S. (1990). Heterogeneity in the Distribution and Morphology of Microglia in the Normal Adult Mouse Brain. Neuroscience.

[65] Streit W.J., Walter S.A., Pennell N.A. (1999). Reactive Microgliosis. Prog. Neurobiol..

[66] Arshadi C., Günther U., Eddison M., Harrington K.I.S., Ferreira T.A. (2021). SNT: A Unifying Toolbox for Quantification of Neuronal Anatomy. Nat. Methods.

[67] Green T.R.F., Murphy S.M., Rowe R.K. (2022). Comparisons of Quantitative Approaches for Assessing Microglial Morphology Reveal Inconsistencies, Ecological Fallacy, and a Need for Standardization. Sci. Rep..

[68] Davis B.M., Salinas-Navarro M., Cordeiro M.F., Moons L., De Groef L. (2017). Characterizing Microglia Activation: A Spatial Statistics Approach to Maximize Information Extraction. Sci. Rep..

