# Supplementary material for: Traumatic Brain Injury Induces Microglial and Caspase3 Activation in the Retina
Source: Int J Mol Sci. 2023 Feb 23;24(5):4451. doi: 10.3390/ijms24054451 (PMC10003323; doi:10.3390/ijms24054451)
Supplement: Supplementary file 1 [file ijms-24-04451-s001.zip › ijms-2168267-supplementary.pdf]

International Journal of
Molecular Sciences

MDPI

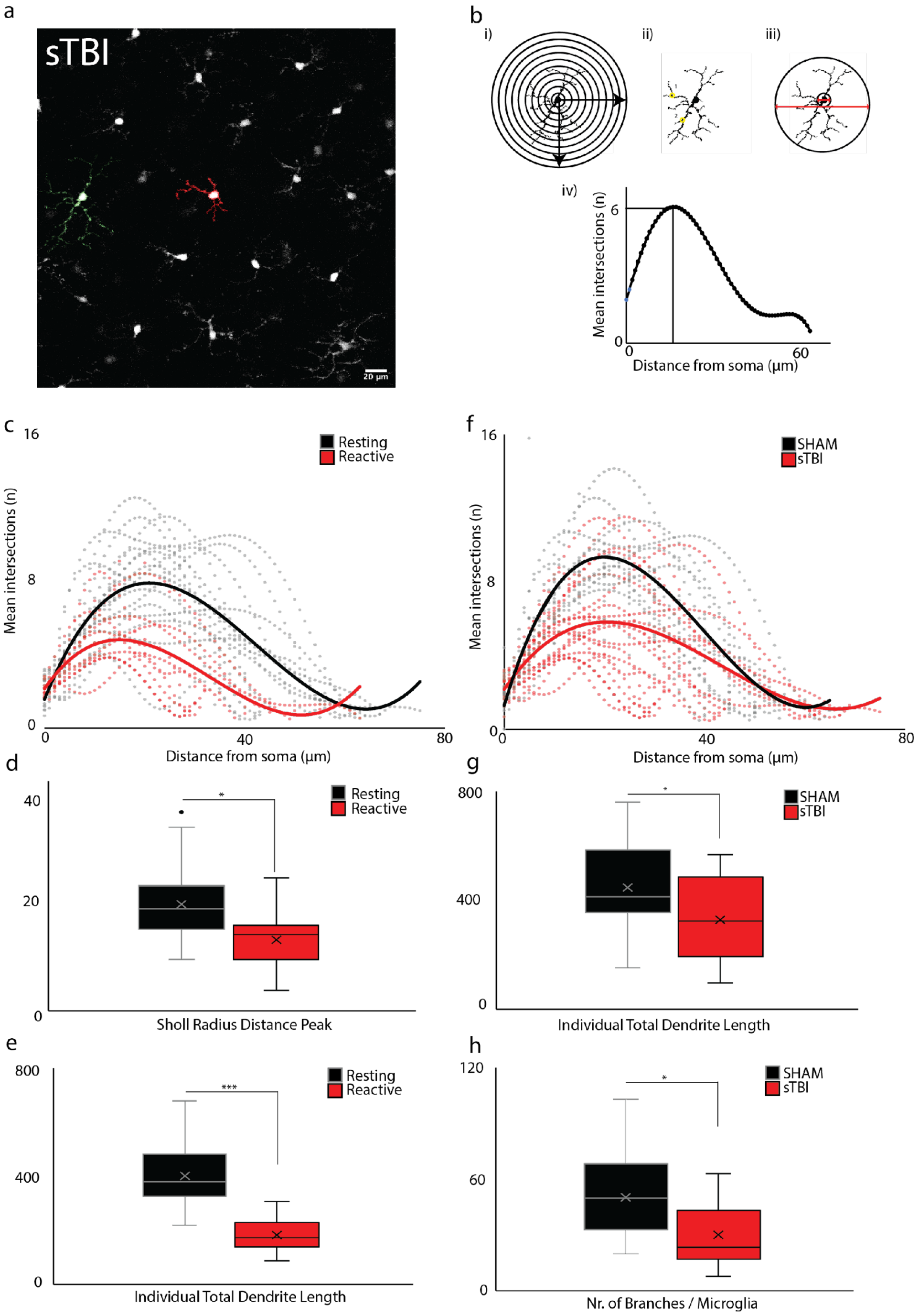

Figure S1. Microglial morphological classification. Panel (a) shows an example sTBI confocal z-merge, with iba1 (gray) IHC labeling all MGs in the field with resting and reactive morphologies, examples of which can be seen with green and red, respectively, created from FIJI SNT reconstructions. In order to distinguish between resting and reactive states, morphological analyses were performed and certain features (b) were evaluated: (i) intersection numbers in bins of Sholl radii, (bin size: 1 µm), (ii) number of branches, total number, and lengths of dendrites in a given microglia, (iii) ratio of soma area/dendritic field area, lastly (iv) the x (distance from the soma) and y (number of intersections at that given distance bin) values were determined at the peak of the Sholl analysis curve. These parameters were utilized to verify the choices made by our trained observers; panel (c) shows Sholl analysis curves of cells previously determined to be resting (black) or reactivated (red) by a trained observer, with a polynomial fit of the order 3 shown (thick line). The reactivated microglia mean curve is shifted towards 0 on both axes, indicating significantly fewer and shorter dendrites, which is indeed a representative morphology of a reactivated

microglia. This change can also be seen from the decrease in the Sholl curve's peak distance ((d), <0.05, *t*-test) and from a substantial (<0.001, *t*-test) decrease in total dendrite length per microglia (e). Panel (f) shows the Sholl analysis values and the 3rd degree polynomial fit curve of MGs (n = 40) from the DL of SHAM treatment (black) retinas and from the DL of sTBI treatment (red) retinas (n = 4) with a decrease in the mean number of intersections. This change indicates a shift towards MG reactivation, which is confirmed by the decrease in individual total dendrite length of the measured MGs ((g), <0.05, *t*-test) and the decrease in the number of branches per MG ((h), <0.05, *t*-test). These data (f–h) verify the significant activation of MGs in the DL of sTBI treatment retinas. (scale bar: 20 μm, *** <0.001, * <0.05);

Table S1. Microglial activation by sTBI and rmTBI. The mean activated number and standard deviation (SD) are indicated.

	**SL-NA**			**SL-Act**			**SL-All**		
	**SHAM**	**sTBI**	**rmTBI**	**SHAM**	**sTBI**	**rmTBI**	**SHAM**	**sTBI**	**rmTBI**
**AVG**	45.5	22.0	34.0	47.3	77.4	51.3	92.8	99.4	85.3
**SD**	17.6	20.0	1.7	18.3	18.9	13.6	18.4	6.8	15.0
	**DL-NA**			**DL-Act**			**DL-All**		
	**SHAM**	**sTBI**	**rmTBI**	**SHAM**	**sTBI**	**rmTBI**	**SHAM**	**sTBI**	**rmTBI**
**AVG**	62.5	21.2	26.0	10.8	46.6	41.0	73.3	67.8	67.0
**SD**	10.1	10.4	13.1	4.6	13.4	11.4	12.1	4.5	14.8

Table S2. Microglial mean number of cells identified as act-Casp3 in TBI treatments in superficial retinal layers (NFL + GCL), indicating sample standard deviation (SD).

**Superficial Layer**	**SHAM**	**sTBI**	**rmTBI**
act-Casp3 cells	15.50	165.20	117.75
SD	1.29	22.45	43.61

Table S3. Average act-Casp3 cell counts in TBI in the deep layers of the retina.

	**SHAM**	**sTBI**	**rmTBI**
**AVG**	**5.0**	**199.5**	**107.8**
SD	4.0	7.5	26.0
fold change		*39.9*	*21.6*
